# Multi‐OMICs orchestration enabled by artificial intelligence in inflammatory bowel disease: An exciting future

**DOI:** 10.1002/ueg2.12663

**Published:** 2024-11-07

**Authors:** Marietta Iacucci, Giovanni Santacroce

**Affiliations:** ^1^ APC Microbiome Ireland College of Medicine and Health University College Cork (UCC) Cork Ireland

**Keywords:** artificial intelligence, inflammatory bowel disease, mult‐OMICs, precision medicine

## INTRODUCTION

Inflammatory bowel disease (IBD) encompasses a spectrum of immune‐mediated disorders of the gastrointestinal tract, driven by the complex and only partially understood host‐microbiome crosstalk.^1^ Individual host features and microbiome composition are central to IBD pathophysiology, influencing disease phenotype, progression, and outcomes. Recent research has demonstrated that host gene expression and microbiota composition are highly personalised, reflecting tissue specificity, disease subtype, and inflammatory status.^1^ Navigating this complexity is crucial for identifying novel therapeutic targets and understanding the mechanisms underlying treatment response, paving the way for precision medicine in IBD.

## MULTI‐OMICs TO DECIPHER IBD

OMIC techniques, including genomics, transcriptomics, proteomics, and metagenomics, are high‐throughput techniques which enable the deep characterisation of IBD patients.^2^ Recently, additional OMICs fields, for example, epigenomics, metabolomics, and lipidomics, have been introduced, expanding our understanding of IBD pathobiology complexity. When applied to biological samples from IBD patients, these techniques allow for patient‐specific characterisation of the disease, uncovering new biological networks and therapeutic targets, and predicting disease progression and outcomes.^2^ Nonetheless, these techniques are mainly conducted separately from each other. Since IBD arises from the convergence of multiple factors, the functional integration of multi‐OMICs data is the key to fully understanding and dissecting the disease.

The literature on multi‐OMICs application in IBD remains scant, though preliminary findings are particularly promising.^3^ Multi‐OMICs approaches have recently shed light on the role of gut microbial ecosystems in IBD,^4^ identifying while also elucidating host‐microbial molecular networks. Additionally, the integration of epigenomic, transcriptomic, and metagenomic data has demonstrated potential in predicting disease outcomes, including the need for biologic therapy.^5^ These encouraging results have led to the foundation of IBD consortia dedicated to leveraging multi‐OMICs data for in‐depth disease characterisation and prediction of treatment responses.^6^ Results from these multi‐centre studies are expected to improve quality of life, minimise patient risk, and reduce expenditures on ineffective treatment.

## ARTIFICIAL INTELLIGENCE‐ENABLED MULTI‐OMICs: SHAPING THE FUTURE OF PRECISION MEDICINE IN IBD

The application of OMICs and multi‐OMICs is constrained by several critical challenges, including the need for specialised expertise in data analysis and interpretation, substantial heterogeneity across studies, and the overwhelming volume of high‐throughput data, which is difficult for humans to manage effectively. Artificial intelligence (AI) offers a transformative opportunity in this context. Applying machine learning models to big data and large digital medical datasets can standardise and accurately integrate these techniques, generating deeper and more comprehensive insights while making the results manageable and practical for clinical use.

In this United European Gastroenterology, Cannarozzi and colleagues provide a comprehensive review of the advancements in AI models applied to OMICs in IBD.^7^ Their analysis underscores the early yet promising applications of AI‐driven multi‐OMICs integration in IBD research. While initial studies have been limited by small sample sizes and less stringent machine learning approaches, the potential for AI to integrate multi‐OMICs data is particularly compelling. One of the most exciting developments is using AI‐enabled multi‐OMICs to characterise host‐microbe interactions and predict responses to therapies accurately. Considering the expanding range of available therapies and the resulting complex therapeutic decision‐making in IBD,^8^ this advancement represents a significant leap forward in precision medicine. AI may enable accurate treatment tailoring to individual patients based on their unique molecular profiles. This personalised approach promises to improve patient outcomes, streamline medical decision‐making, and reduce healthcare costs by optimising therapeutic strategies and dosages.

AI can push the boundaries further by enabling a more holistic approach that integrates diverse patient data, including clinical records, laboratory results, endoscopic findings, histologic features, and multi‐OMICs. This multi‐modal integrative approach, known as ‘endo‐histo‐omics’,^9^ represents the most comprehensive approach to patient profiling, shaping the future of precision medicine in IBD. It holds the promise to bridge long‐standing gaps in IBD clinical trials and clinical practice, such as early diagnosis, biomarker discovery, outcome prediction, and personalised treatment decisions. Recent advancements in foundation models are pivotal to achieving this goal.^10^ These models, pre‐trained on extensive and diverse datasets and then fine‐tuned for specific tasks, excel at seamlessly integrating and analysing multimodal datasets, potentially expediting the implementation of ‘endo‐histo‐omics’ in clinical settings.

## CONCLUSION

Despite ongoing challenges in clinical implementation, such as data quality, reproducibility, explainability, trustworthiness, data privacy, and cyber‐security, AI offers a novel, accurate and standardised integration of multi‐OMICs with clinical, endoscopic and histological data. This integration have the potential to translate innovative research into practical advancements in personalised patient care in IBD.

## CONFLICT OF INTEREST STATEMENT

The authors declare no conflict of interest related to this editorial.

## Data Availability

Data sharing is not applicable to this article as no new data were created or analyzed in this study.
